# Manipulating the growth environment through co-culture to enhance stress tolerance and viability of probiotic strains in the gastrointestinal tract

**DOI:** 10.1128/aem.01502-23

**Published:** 2023-11-29

**Authors:** Kosuke Oana, Kensuke Shimizu, Toshihiko Takada, Hiroshi Makino, Mikiko Yamazaki, Miyuki Katto, Minoru Ando, Takashi Kurakawa, Kenji Oishi

**Affiliations:** 1 Basic Research Department, Yakult Central Institute, Tokyo, Japan; 2 Food Research Department, Yakult Central Institute, Tokyo, Japan; 3 Safety Research Department, Yakult Central Institute, Tokyo, Japan; 4 Research Management Center, Yakult Central Institute, Tokyo, Japan; Universita degli Studi di Napoli Federico II, Portici, Naples, Italy

**Keywords:** lactic acid bacteria, probiotics, stress tolerance, biotechnology

## Abstract

The viability of probiotics in the gastrointestinal tract is essential because it has an important role in their health benefits. Plasticity is a phenomenon associated with stress tolerance in bacteria, and stress tolerance of probiotic strains can be induced by the use of additives and weak stress. However, some of these processes are difficult to implement in the manufacture of probiotic beverages, and there are few methods that can be easily applied. Co-culture techniques have been used to produce fermented beverages and may impart distinct characteristics through differences in the culture environment compared with those of monoculture. Here, we show that the stress tolerance of a probiotic strain was altered by co-culture with other strains already used in product manufacturing. Stress tolerance in *Lacticaseibacillus paracasei* strain Shirota was enhanced by co-culture with *Lactococcus lactis* subsp. *lactis* YIT 2027 as a result of an earlier pH decrease. Therefore, the stress tolerance was similarly improved in monocultures where pH was manipulated. Strains with improved stress tolerance had an increase in dihydrosterculic acid in the cell membrane, with altered gene transcription in energy metabolism, lipid metabolism, and chaperone genes. Moreover, fermented beverage consumption studies revealed that the probiotics produced through co-culture were significantly more viable in the human gastrointestinal tract than those produced through monoculture. These data suggested that co-culture with specific strains can significantly enhance the viability of probiotic strains in the human gastrointestinal tract.

**IMPORTANCE:**

The viability of probiotics in the human gastrointestinal tract is important, as some reports indicate that the health benefits of live bacteria are greater than those of dead ones. Therefore, the higher the viability of the probiotic strain, the better it may be. However, probiotic strains lose their viability due to gastrointestinal stress such as gastric acid and bile. This study provides an example of the use of co-culture or pH-controlled monoculture, which uses more stringent conditions (lower pH) than normal monoculture to produce probiotic strains that are more resistant to gastrointestinal stress. In addition, co-cultured beverages showed higher viability of the probiotic strain in the human gastrointestinal tract than monocultured beverages in our human study.

## INTRODUCTION

Today, many kinds of probiotics are commercially available. The original term “probiotic” was intended to refer to substances that were the opposite of “antibiotic” (1). There is a widely accepted definition of probiotics, as stated by the British microbiologist Fuller in 1989, as “a live microbial feed supplement which beneficially affects the host animal by improving its intestinal microbial balance” (2). Additionally, in 2001, the Food and Agriculture Organization of the United Nations and the World Health Organization published a definition of probiotics as “live microorganisms which when administered in adequate amounts confer a health benefit on the host” (3). *Lacticaseibacillus paracasei* strain Shirota (LcS), formerly *Lactobacillus casei* strain Shirota, is a well-established probiotic organism. There are numerous reported health benefits associated with its consumption, including host defense against infection (4), improvement of gastrointestinal symptoms (5, 6), immuno-enhancing effect (7), control of upper respiratory tract infection (8), and improvement of sleep quality (9). The question of whether the viability of probiotic strains is necessary for their health benefits remains unresolved, but current evidence suggests that viable strains often produce more extensive biological responses than non-viable strains (10). Viable strains can regulate the gut microbiome and also affect the immune system, whereas non-viable strains primarily act on the immune system (11). Structured bacterial molecules (e.g., bacterial cell walls and lipoteichoic acids) are important for activating the immune system and are found in both live and dead probiotic strains (12). However, the improvement of the intestinal environment, such as the elimination of pathogens, is thought to involve the biological activity of live bacteria (13).

If probiotic strains that are alive at the time of consumption lose their viability in the gastrointestinal tract, then their consumption is considered equivalent to consuming dead bacteria. The viability of probiotic strains can be greatly influenced by various factors in the gastrointestinal tract, such as gastric acid, bile, and digestive enzymes, and these influences can reduce their viability (14). For probiotic strains to exert their physiological effects in a live state on the host, they need to withstand the harsh environment of the gastrointestinal tract. In the context of these circumstances, research has been performed to investigate the stress tolerance of probiotic strains (15). Studies have employed simulated gastric acid and bile to identify probiotic strains with elevated viability (16), and additional research has been conducted to uncover methods and mechanisms to enhance viability (17).

Three main methods have been considered to increase the viability of bacteria (18): using additives in the culture medium, inducing adaptive responses, and encapsulation. First, adding fatty acids, such as oleic acid, to the culture medium modulates the fatty acid composition of the bacteria and increases their resistance to stress (19). Second, many bacteria possess adaptive mechanisms that allow them to survive lethal stressors after prior exposure to non-lethal stressors (20). Research has been conducted by using various forms of mild stress stimuli, including heat, pH changes, exposure to ethanol, and the presence of salt, to induce adaptive responses and enhance the stress tolerance of probiotic strains (21). Third, encapsulation has been linked to recent technological advances, and a variety of capsules have been studied (22). Nevertheless, despite extensive research into improving the viability of probiotic strains, there are several challenges to overcome. For example, the safety of fatty acids has been verified by the US Food and Drug Administration, but alteration of the gut microbiome by these substances has been documented (23, 24). In addition, using stress stimuli to induce adaptive responses in the production of a probiotic beverage is difficult owing to the complexity of the manufacturing process (25). Furthermore, encapsulation has been applied to manufacturing only in a few cases (26). Therefore, we aimed to develop a novel method that could be applied to manufacturing and would enhance the viability of probiotic strains. Giving the probiotic strains high viability will allow us to verify the health benefits of more viable bacteria passing through the gastrointestinal tract. It is expected that such verification will provide a better understanding of the sufficient dose of viable probiotic strains.

Some probiotic beverages are produced by the monoculture of probiotic strains, whereas others are produced by co-culture with other strains. Considering that the culture environment affects the stress tolerance of bacteria (18), we hypothesized that co-culture would lead to different culture environments and result in different stress tolerances than would occur with monoculture. Here, we compared the stress tolerance of a representative probiotic strain, LcS, cultured under monoculture and co-culture conditions. By using simulated gastric acids and bile, we assessed the variations in stress tolerance of LcS and investigated the factors underlying these differences. Finally, differences in viability in the human gastrointestinal tract were examined by the consumption of LcS-fermented beverages produced by monoculture and by co-culture.

## RESULTS

### Co-culture enhances the tolerance of *L. paracasei* to simulated gastrointestinal stress

**Fig 1:**
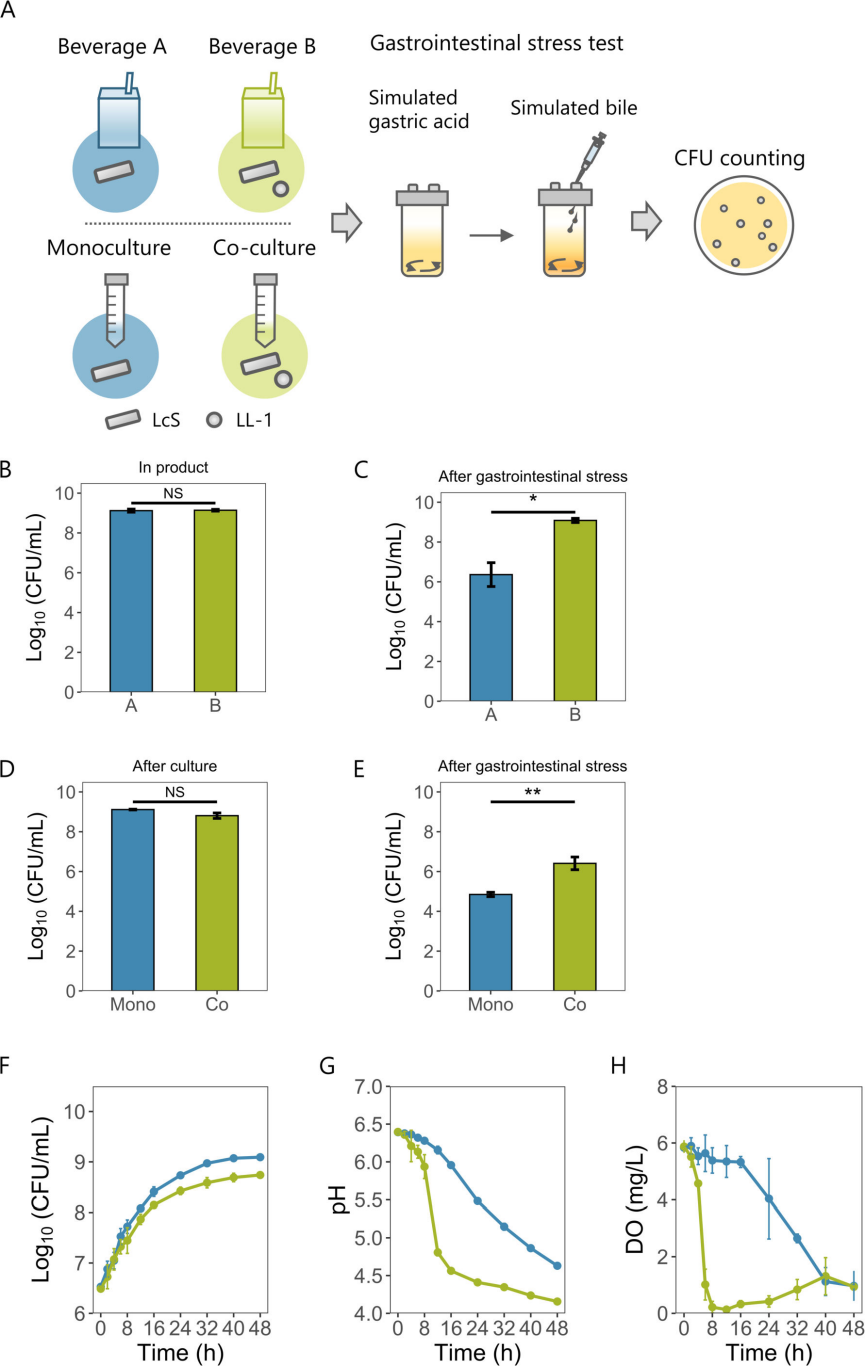
Comparison between LcS monoculture and co-culture with LL-1. (**A**) Simulated gastrointestinal stress test procedure. Monocultured or co-cultured fermented milk products or bacterial cultures were added to pH-4.0 simulated gastric acid. Bacteria were incubated for 1 h and then the pH was raised to 4.4, after which they were incubated with 0.3% oxgall for 5 min. Following the incubation, serial dilutions were plated onto lactitol-LBS-vancomycin (LLV) agar and CFUs were counted. (**B**) Log_10_ (CFU/mL) of LcS in each beverage (mean ± SD, *n* = 3). (**C**) Log_10_ (CFU/mL) of LcS in each beverage after simulated gastrointestinal stress (mean ± SD, *n* = 3). (**D**) Log_10_ (CFU/mL) of LcS after each culture (mean ± SD, *n* = 3). (**E**) Log_10_ (CFU/mL) of LcS after simulated gastrointestinal stress (mean ± SD, *n* = 3). (**F–H**) Log_10_ (CFU/mL) (**F**), pH (**G**), and DO (**H**) changes during both cultures (mean ± SD, *n* = 3). Blue lines indicate monoculture and green lines indicate co-culture. (B–E) Statistical significances were determined by using a two-tailed Welch’s *t*-test (**P < 0.01; *P < 0.05; and NS, not significant).

Among the fermented milk products containing LcS, there exists a probiotic beverage that is co-cultured with *Lactococcus lactis* subsp. *lactis* YIT 2027 (LL-1). To compare the stress tolerance of LcS in products produced by monoculture (Beverage A) and co-culture (Beverage B) or in bacterial solutions cultured by using these methods, a simulated gastrointestinal stress test, a gastric acid-bile continuous stress test, was performed (Fig. 1A). Although the LcS CFU densities in the two products were similar (Fig. 1B), Beverage B had a significantly higher CFU density after simulated gastrointestinal stress (Fig. 1C). Next, to compare the stress tolerance more rigorously, the same milk media and incubation conditions were used in the two cultures, as the culture media and conditions used routinely in product manufacturing differ. There was no significant difference in LcS CFU densities after 48 h of culture (Fig. 1D), but co-cultured LcS had significantly higher CFU densities than monocultured LcS after simulated gastrointestinal stress (Fig. 1E). The LcS CFU densities in both cultures increased similarly over time (Fig. 1F), but co-culture gave a drastic decrease in pH after 12 h of culture (Fig. 1G) and in dissolved oxygen (DO) after 6 h (Fig. 1H). The increase in LL-1 was greater than that in LcS at an early culture phase (Fig. S1). Since *L. lactis* produces lactic acid and consumes oxygen as it grows (27), the early increase in LL-1 was thought to cause a decrease in pH and DO.

### Stress tolerance is improved in pH-controlled monoculture mimicking co-culture pH

**Fig 2:**
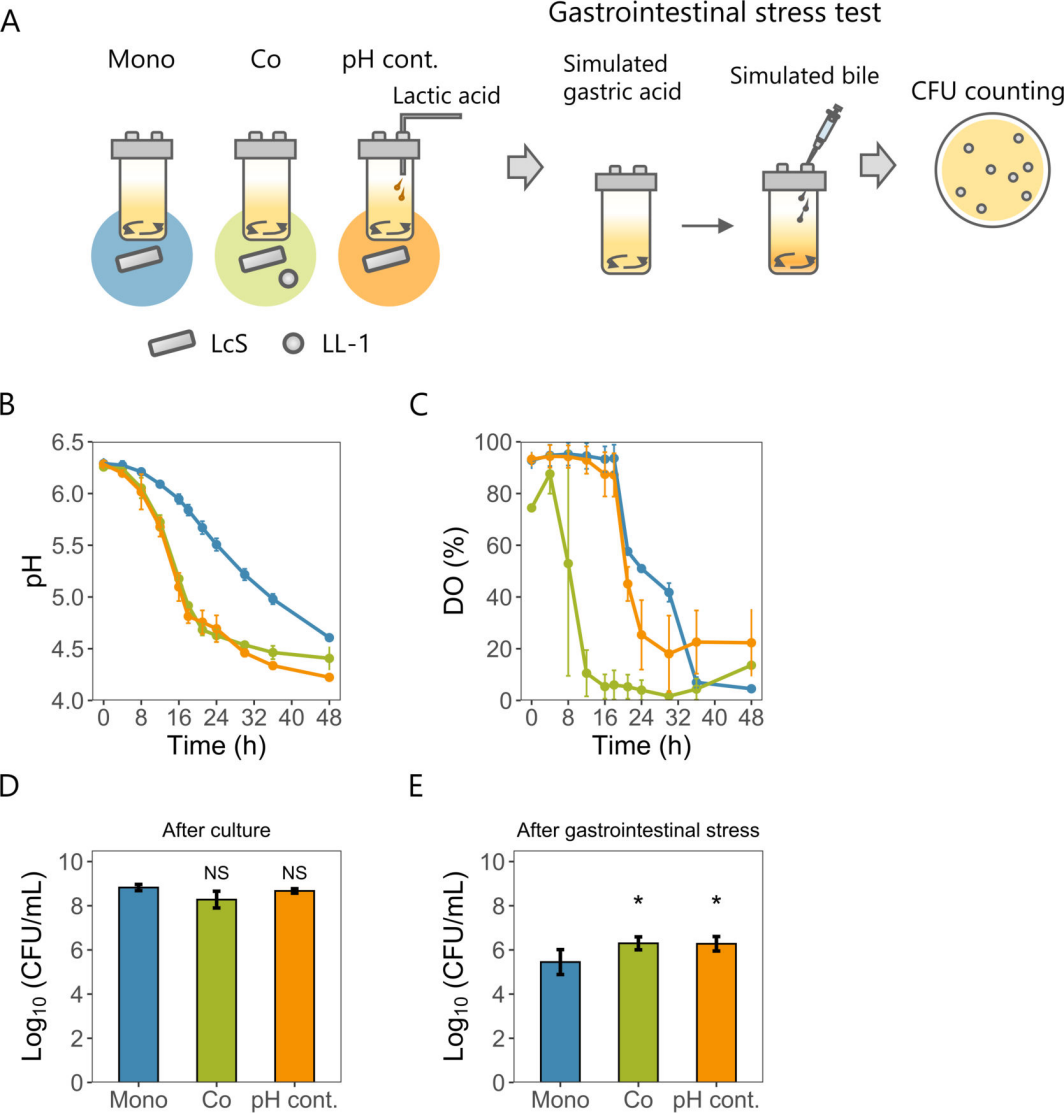
Comparison among LcS monoculture, LcS co-culture with LL-1, and pH-controlled LcS monoculture. (**A**) Procedure for jar culture and simulated gastrointestinal stress test. Lactic acid was used for pH control in the pH-controlled monoculture. The simulated gastrointestinal stress tests were performed in a similar manner as shown in Fig. 1A. (**B**) pH changes under the three culture conditions (mean ± SD, *n* = 3). (**C**) DO changes under the three culture conditions (mean ± SD, *n* = 3). (**B** and **C**) Blue lines indicate monoculture, green lines indicate co-culture, and orange lines indicate pH-controlled monoculture. (**D**) Log_10_ (CFU/mL) of LcS after each culture (mean ± SD, *n* = 3). (**E**) Log_10_ (CFU/mL) of LcS after simulated gastrointestinal stress (mean ± SD, *n* = 4). (D–E) Statistical significances relative to monoculture were determined by using one-way analysis of variance (ANOVA) with Dunnett’s multiple comparison test (*P < 0.05 and NS, not significant).

To test the hypothesis that anaerobic or low pH conditions of co-culture improve stress tolerance, we first investigated whether an anaerobic environment contributed to enhanced stress tolerance. Anaerobically cultured LcS did not show enhanced stress tolerance after the simulated gastric acid challenge (Fig. S2). To confirm that the pH fluctuations in co-culture enhanced stress tolerance, a pH-controlled monoculture was designed to mimic the co-culture pH. The stress tolerance of LcS was then compared among the monoculture, co-culture, and pH-controlled monoculture (Fig. 2A). The pH-controlled monoculture mimicked the pH change of the co-culture (Fig. 2B), although the pattern of DO change in the pH-controlled monoculture differed from that of the co-culture (Fig. 2C). The LcS CFU densities after 48 h of culture were similar in all cultures (Fig. 2D). However, the pH-controlled monoculture gave significantly higher LcS CFU densities after simulated gastrointestinal stress than the monoculture (Fig. 2E), similar to the results obtained with the co-culture (Fig. 1E). These results indicated that culture pH was crucial for enhancing stress tolerance.

### Other combination of strains also gives improved stress tolerance

**Fig 3:**
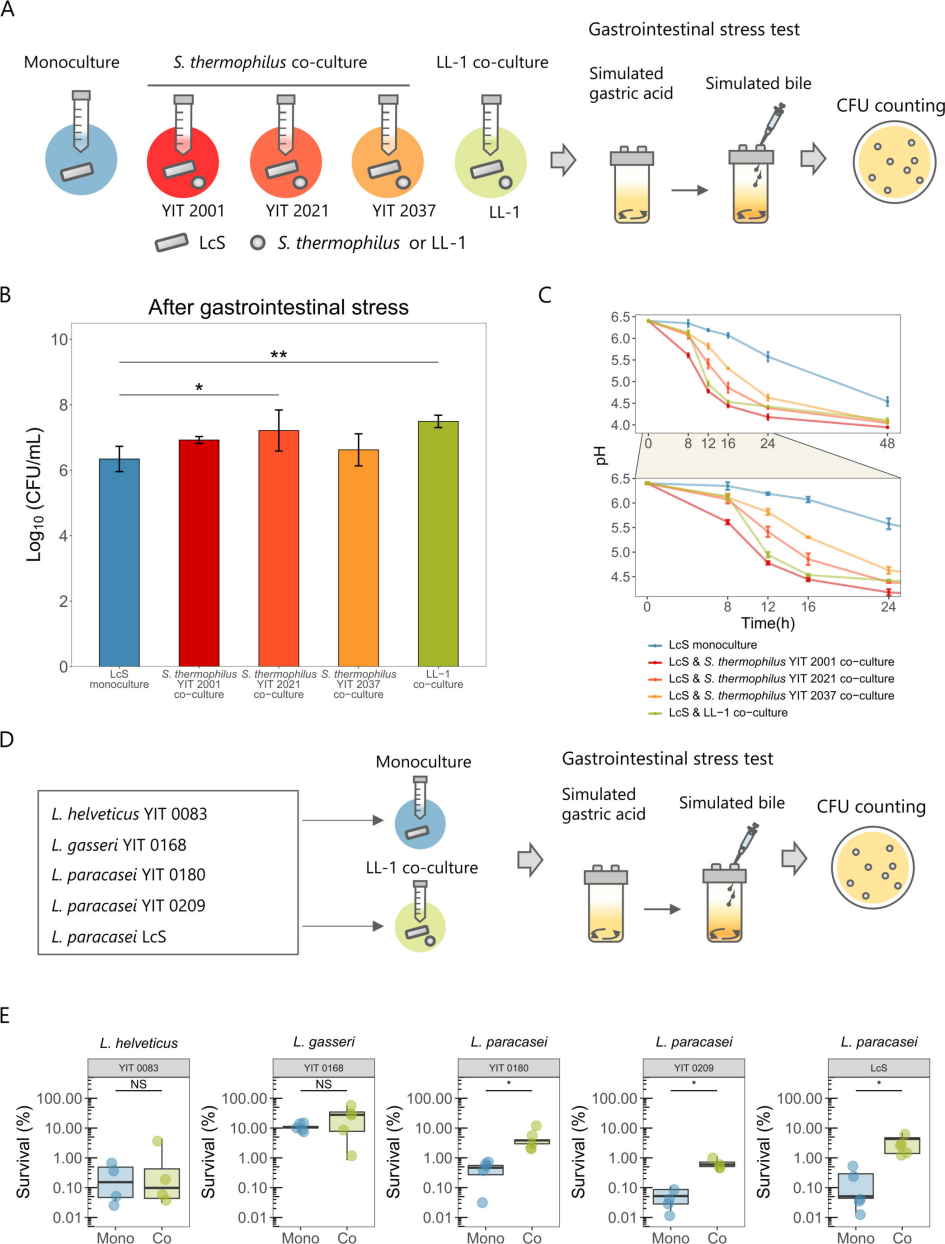
Stress tolerance through co-culture in various combinations. (**A**) LcS was co-cultured with *Streptococcus thermophilus* YIT 2001, YIT 2021, or YIT 2037 in addition to being monocultured or co-cultured with LL-1. After 48 h of culture, a simulated gastrointestinal stress test was performed. (**B**) Log_10_ (CFU/mL) of LcS after simulated gastrointestinal stress (mean ± SD, *n* = 4). (**C**) pH changes under the five culture conditions (mean ± SD, *n* = 3). The lower panel is an enlarged view of the 0- to 24-h period. (**D**) In addition to LcS, *Lactobacillus helveticus* YIT 0083, *Lactobacillus gasseri* YIT 0168, *Lacticaseibacillus paracasei* YIT 0180, or *Lacticaseibacillus paracasei* YIT 0209 were monocultured or co-cultured with LL-1. After 48 h of culture, a simulated gastrointestinal stress test was performed. Simulated gastric acid at pH 4.0 was used for all strains, and oxgall concentrations of 0.16% (YIT 0083 and YIT 0209) and 0.30% (YIT 0168, YIT 0180, and LcS) were used as simulated bile. (**E**) Percentage of bacteria surviving after the simulated gastrointestinal stress in each strain (mean ± SD, *n* = 4 or 5). (**B**) Statistical significances relative to LcS monoculture were determined by using one-way ANOVA with Dunnett’s multiple comparison test (**P < 0.01 and *P < 0.05). (**E**) Statistical significances were determined by using two-tailed Welch’s *t*-test (*P < 0.05 and NS, not significant).

Next, we investigated whether co-culturing LcS with lactic acid bacteria strains other than LL-1 would improve stress tolerance. Simulated gastrointestinal stress was examined upon co-culture of LcS with three strains of *Streptococcus thermophilus*, which grows as well on milk media as LL-1 (Fig. 3A). The stress tolerance of LcS was significantly enhanced in co-culture with *S. thermophilus* YIT 2021 (Fig. 3B). Co-cultures with these strains gave an earlier decrease in pH than did LcS monoculture, although each pH fluctuation was different (Fig. 3C). The pH in co-culture with *S. thermophilus* YIT 2001 decreased earlier than that in LL-1 (mean pH 5.61 at 8 h vs LL-1 pH 6.13). In contrast, in co-culture with *S. thermophilus* YIT 2037, the pH decreased later than that in LL-1 (mean pH 5.30 at 16 h vs LL-1 pH 4.53). Calculation of the dissimilarity of the pH fluctuations revealed that the pH change in *S. thermophilus* YIT 2021 co-culture was the most similar to that in LL-1 co-culture (Fig. S3). This supported the hypothesis that pH fluctuation regulates LcS stress tolerance. Other *Lactobacilli* strains were also examined to assess whether co-culture with LL-1 would enhance their stress resistance (Fig. 3D). Co-culture with LL-1 significantly improved stress tolerance in the *L. paracasei* strains, but not in *Lactobacillus gasseri* or *Lactobacillus helveticus* (Fig. 3E).

### Increased dihydrosterculic acid content of cell membrane improves stress tolerance

**Fig 4:**
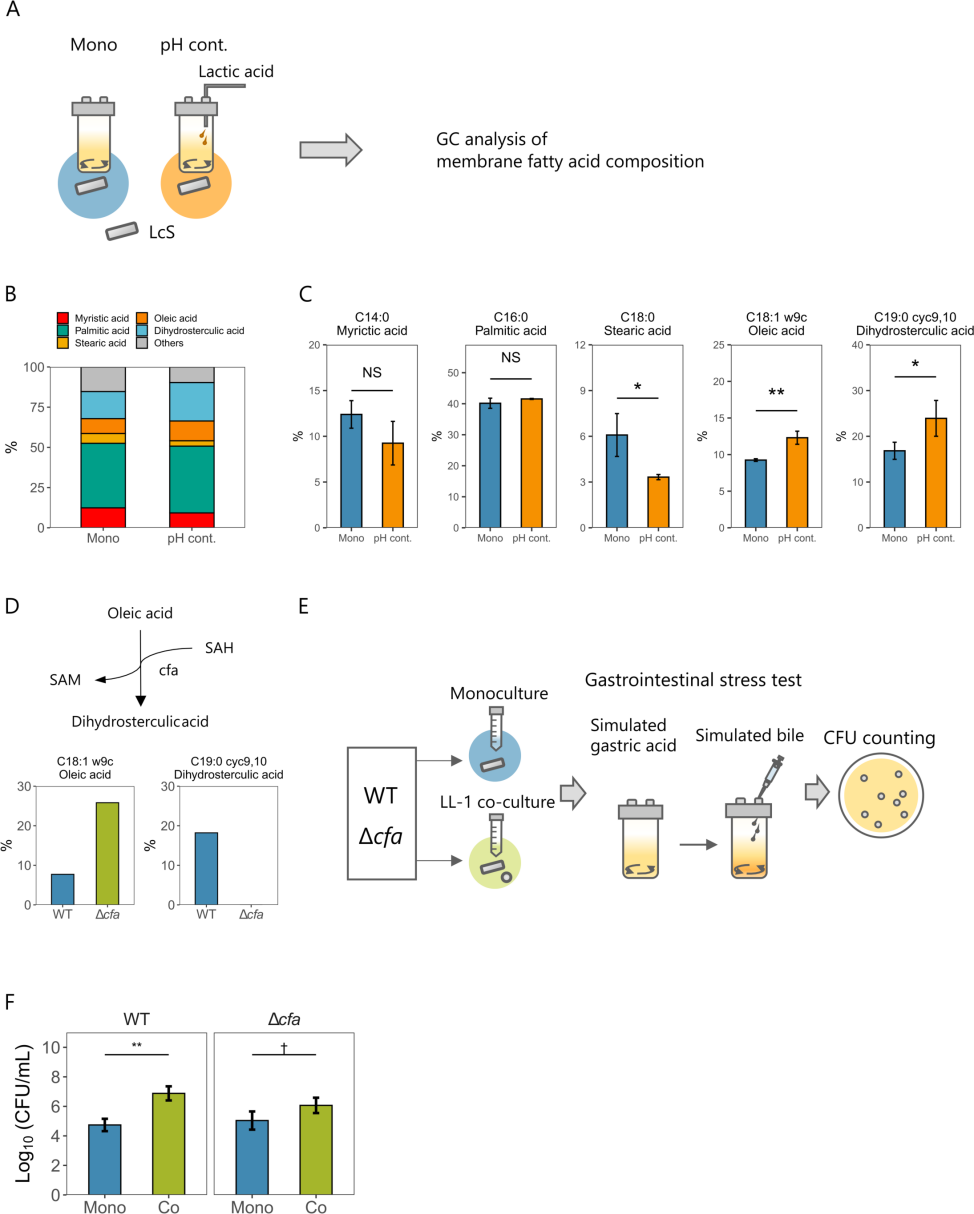
Relationship between culture-induced changes in cell-membrane fatty acid composition and stress tolerance of LcS. (**A**) After 48 h of culture, LcS in both monoculture and pH-controlled monoculture were harvested and analyzed for their cell-membrane fatty acid composition by gas chromatography (GC). (**B**) Major cell-membrane fatty acid compositions of LcS in each culture (mean, *n* = 3). (**C**) Comparison of cell-membrane fatty acid compositions of LcS in each culture (mean ± SD, *n* = 3). (**D**) Enzymatic reaction of dihydrosterculic acid and comparison of oleic acid and dihydrosterculic acid compositions in LcS wild-type (WT) and *cfa*-deficient (Δ*cfa*) strains (*n* = 1). SAM, *S*-adenosylmethionine; SAH, *S*-adenosylhomocysteine. (**E**) Procedures used to examine simulated gastrointestinal stress resistance of WT and Δ*cfa* strains in monoculture and co-culture. (**F**) Log_10_ (CFU/mL) of LcS after simulated gastrointestinal stress in WT and Δ*cfa* strains in monoculture and co-culture (mean ± SD, *n* = 3). (**C**) Statistical significances were determined by using two-tailed Student’s *t*-test (**P < 0.01; *P < 0.05; and NS, not significant). (**F**) Statistical significances were determined by using a two-tailed Welch’s *t*-test (**P < 0.01; †*P* < 0.1).

The cell-membrane fatty acid composition is known to change with the culture environment and affects stress tolerance (28). Owing to the difficulty of separating the two strains in co-culture, we compared the cell-membrane fatty acid composition of LcS between the monoculture and the pH-controlled monoculture (Fig. 4A; Fig. S4A). The major cell-membrane fatty acids of LcS in both cultures were palmitic acid, dihydrosterculic acid, myristic acid, oleic acid, and stearic acid (Fig. 4B). The content of stearic acid was significantly reduced in the pH-controlled monoculture, whereas those of oleic acid and dihydrosterculic acid were significantly increased (Fig. 4C). Dihydrosterculic acid (a cyclopropane-type fatty acid) enhances stress tolerance in some bacterial species (29, 30), and it is converted from oleic acid by a cyclopropane-type fatty acid synthase encoded by *cfa* (31). LcS has one *cfa* gene (DDBJ/EMBL/GenBank accession number: LC771591) in its genome. Therefore, we created a *cfa*-deficient strain to investigate whether increased dihydrosterculic acid by co-culture contributes to stress tolerance. The *cfa*-deficient strain had no detectable dihydrosterculic acid but had an increased content of its precursor, oleic acid (Fig. 4D; Fig. S4B). The wild-type and *cfa*-deficient strains were then monocultured or co-cultured with LL-1 to compare their stress tolerance (Fig. 4E). Unlike the wild-type strain, the *cfa*-deficient strain in co-culture with LL-1 had no significant enhancement of stress tolerance (Fig. 4F). These data suggested that an increased dihydrosterculic acid content by co-culture contributed to improved stress tolerance. Moreover (although the result was not significant), co-cultured *cfa*-deficient strain tended to have a higher LcS CFU density than the monocultured one, suggesting the presence of another adaptive mechanism.

### Transcriptome analysis reveals the genes involved in stress tolerance

**Fig 5:**
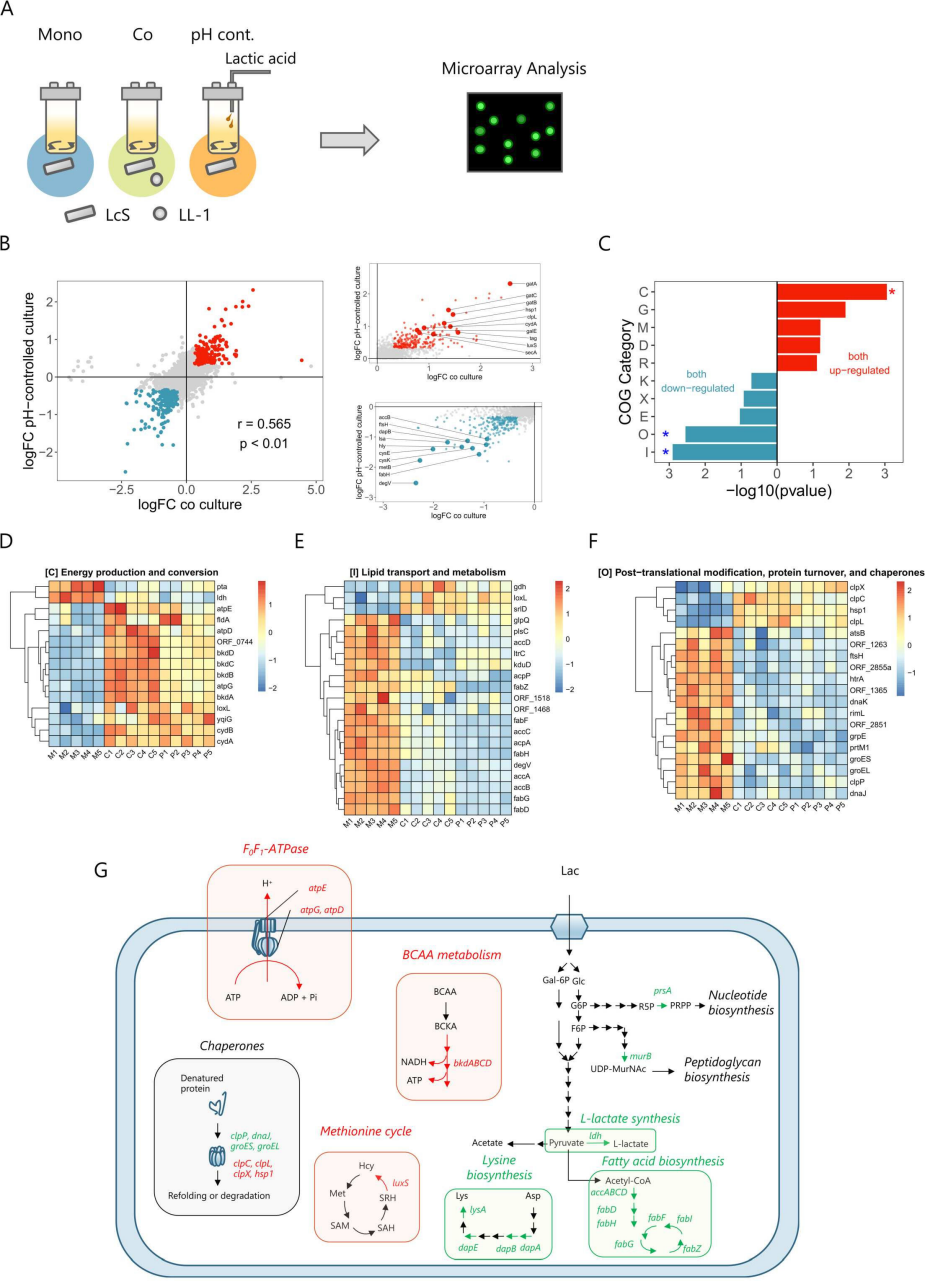
Transcriptome analysis of LcS in monoculture, co-culture, and pH-controlled monoculture. (**A**) RNA was extracted from each culture after 48 h of growth, and transcriptome analysis was conducted by using microarray technology (*n* = 5). (**B**) The log_2_ fold change (logFC) of each gene was determined in both co-culture and pH-controlled monoculture, relative to that in monoculture. Genes with Benjamini–Hochberg adjusted *P-*values (adj. *P*) >0.05 are indicated in gray. Among genes with adj. *P* <0.05, those with logFC > log_2_ (1.25) are indicated in red, whereas those with logFC < -log_2_ (1.25) are indicated in blue. The top and bottom 10 annotated genes are presented in the enlarged panels. Pearson’s correlation coefficient between co-culture and pH-controlled culture was 0.565. (**C**) COG enrichment analysis of genes commonly expressed at high or low levels in co-culture and pH-controlled monoculture. Asterisks indicate COG categories with Benjamini–Hochberg adjusted *P*-values < 0.05. (D–F) Levels of expression of genes in COG categories C (energy production and conversion), I (lipid transport and metabolism), and O (post-translational modification, protein turnover, and chaperones), which were commonly found to be expressed at higher or lower levels in both co-culture and pH-controlled monoculture compared to the monoculture . M1–M5: monoculture; C1–C5: co-culture; and P1–P5: pH-controlled monoculture. (**G**) Inference of alterations in gene function in both co-culture and pH-controlled monoculture, as determined by microarray analysis. BCAA, branched-chain amino acid; BCKA, branched-chain α-keto acid; Lac, lactose; Gal-6P, galactose-6-phosphate; Glc, glucose; G6P, glucose-6-phosphate; R5P, ribose 5-phosphate; PRPP, phosphoribosyl pyrophosphate; F6P, fructose 6-phosphate; UDP-MurNAc, uridine diphosphate N-acetylmuramic acid; Hcy, homocysteine; Met, methionine; SAM, *S*-adenosylmethionine; SAH, *S*-adenosylhomocysteine; and SRH, *S*-ribosylhomocysteine.

Microarray analysis was used to investigate the presence of another mechanism behind the improved stress tolerance (Fig. 5A). We found that 243 genes were significantly upregulated, and 250 genes were significantly downregulated in both the co-culture and the pH-controlled monoculture compared with the monoculture (Fig. 5B). The top 10 annotated genes commonly upregulated were *gatA*, *gatC*, *gatB*, *hsp1*, *clpL*, *cydA*, *galE*, *tag*, *luxS*, and *secA*, and the top 10 downregulated genes were *degV*, *fabH*, *metB*, *cysK*, *cysE*, *hly*, *lsa*, *dapB*, *ftsH*, and *accB* (Fig. 5B). Highly expressed genes included *hsp1* and *clpL*, encoding chaperone proteins, and *luxS*, contributing to methionine metabolism and autoinducer-2 production. These genes are involved in stress tolerance in *Lactobacilli* (32–34). Downregulated genes included *dapB*, which is involved in lysine biosynthesis from aspartate. Aspartate has been reported to be involved in stress tolerance (35), and other genes in this pathway were also significantly downregulated (Fig. S5A). Expression of the *cfa* gene did not differ significantly among the three cultures (Fig. S5B), suggesting that factors other than this gene altered the dihydrosterculic acid content of the cell membrane. Enrichment analysis of commonly altered genes by using COG (Clusters of Orthologous Groups) classification (36) revealed the upregulation of genes in COG classification C and downregulation of genes in COG classifications I and O (Fig. 5C). Genes in the COG C classification are involved in energy production and conversion. The expression levels of *atpE*, *atpD*, and *atpG*, which encode subunits of F_0_F_1_-ATPase (37), and of *bkdD*, *bkdC*, *bkdB*, and *bkdA*, which are involved in branched-chain amino acid metabolism (38), were increased (Fig. 5D). In contrast, the expression of *ldh*, which encodes an enzyme converting pyruvate to lactic acid (39), was decreased (Fig. 5D). Among the genes around the glycolytic system, *prsA* and *murB*, which are important for nucleic acid biosynthesis (40) and peptidoglycan biosynthesis (41), respectively, were downregulated (Fig. S5C and D). The cell-wall thickness of LcS in the pH-uncontrolled and pH-controlled monocultures was also evaluated but was not found to differ (Fig. S5E). The COG I classification comprises a group of genes involved in lipid transport and metabolism. The expression of fatty acid synthase genes, including *accA*, *accB*, *accC*, *accD*, *fabD*, *fabH*, *fabG*, *fabZ*, and *fabF*, was decreased (Fig. 5E). The COG O classification is a group of genes associated with post-translational modification, protein turnover, and chaperones. Expression of *ftsH*, *htrA*, *dnaK*, *grpE*, *groES*, *groEL*, *clpP*, and *dnaJ* was decreased, whereas that of *clpX*, *clpC*, *hsp1*, and *clpL* was increased (Fig. 5F). On the basis of these results, we summarized the inferred functions of the genes that were commonly differentially expressed in both co-culture and pH-controlled monoculture (Fig. 5G).

### Probiotic beverages from co-culture show greater viability in the human gastrointestinal tract than those from monoculture

**Fig 6:**
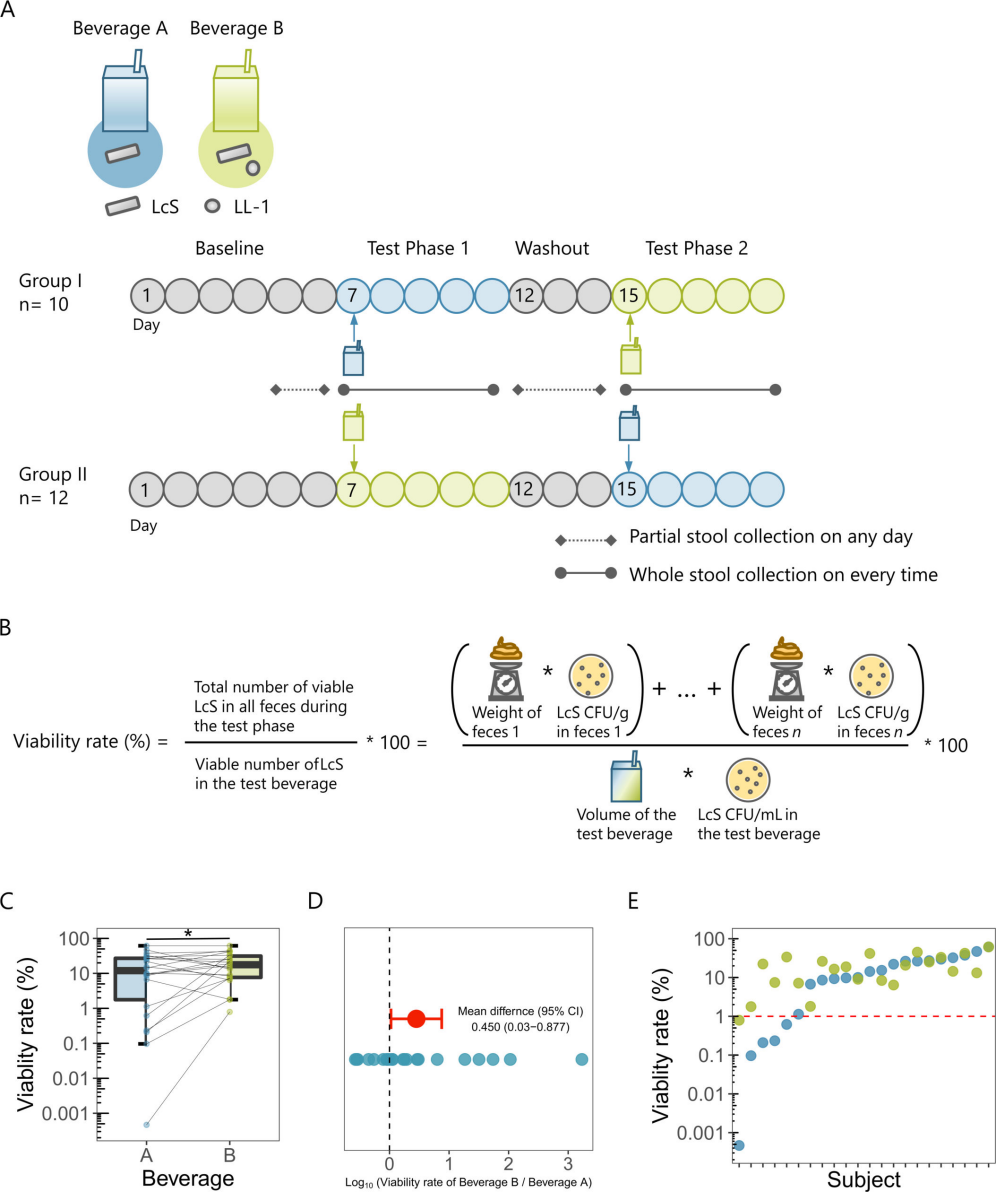
Comparison of numbers of LcS surviving after passing through the human gastrointestinal tract following the consumption of a monocultured or co-cultured LcS beverage. (**A**) Overview of the experiment. Participants were allocated to Groups I and II and underwent testing in a crossover design utilizing varying orders of beverage consumption. On day 7, after the initiation of dietary restriction, a single serving of either Beverage A or B was consumed, and whole-feces samples were collected for all defecations over the subsequent 4 days. After a 3-day washout period, a single serving of either beverage B or A was consumed, and all whole-feces samples were again collected over the next 4 days. A portion of feces was collected on day 5 or 6 and day 12, 13, or 14 before the consumption of each test beverage to ensure that LcS were not detectable. (**B**) Definition of LcS viability rate and its calculation method. LcS viability rate was calculated by dividing the total number of viable LcS in all feces during the test phase by the number of viable LcS in the test beverage and multiplying by 100. The number of viable LcS in the feces or beverages was calculated by multiplying the weight or volume by the LcS CFU density. (**C**) LcS viability rate in feces following a single consumption of each test beverage (*n* = 22). (**D**) Differences in viability rates of LcS between the test beverages. Blue circles indicate the log_10_ (viability rate of Beverage B/viability rate of Beverage A) for each subject, and red circle indicates the mean of these differences, with a 95% confidence interval. (**E**) Viability rate of LcS per test beverage for each subject. The blue circles indicate the viability rate of Beverage A, and the green circles indicate the viability rate of Beverage B. The red dotted line indicates a 1% viability rate. (**C**) Statistical significance was determined by using a two-tailed paired *t*-test (*P < 0.05).

To compare LcS viability in the human gastrointestinal tract between different culture methods, we performed consumption studies by using fermented LcS products derived from monoculture (Beverage A) and from co-culture with LL-1 (Beverage B) (Fig. 6A, see Materials and Methods). Whole feces were collected during the 4 days after consumption to determine the viability rates of ingested LcS in the feces (Fig. 6B). As fecal weight and gastrointestinal transition time varied among the subjects (Fig. S6A), we considered that the viability rate would give a more accurate measure of viability in the gastrointestinal tract than CFU density, although in fact the viability rate and the CFU/g of LcS were highly correlated (Fig. S6B). The results of the consumption test showed that the viability rate of LcS in the feces was significantly higher when the subjects consumed Beverage B (Fig. 6C), at an average of 2.8 (10^0.450^) times higher (Fig. 6D). In particular, five subjects with less than 1% LcS viability when they consumed Beverage A showed large improvements in LcS viability when they consumed Beverage B (Fig. 6E). It is difficult to determine the viability rate of consumed bacteria if they grow in the gastrointestinal tract. Since no LcS cells were lost due to the *in vitro* gastrointestinal stress (Fig. S7A through C), we investigated the total counts of LcS, including viable and non-viable, in fecal samples and beverages. The percentage of total counts of LcS in the feces relative to the beverage was approximately 80% of that in both beverages, and there was no significant difference between them (Fig. S7D). These data suggested that LcS in both beverages are unlikely to grow in the gastrointestinal tract.

## DISCUSSION

The viability of probiotics within the human gastrointestinal tract can be pivotal for their efficacy. Therefore, improving the stress tolerance of these bacteria is an important area of study in applied microbiology. Here, we hypothesized that the stress tolerance of probiotic strains would change with the method of culture, whether it be monoculture or co-culture. Our *in vitro* studies showed that co-culture enhanced stress tolerance, which was attributable to a reduction of pH in the early stages of culture. Additionally, the composition of fatty acids in the cell membrane was found to be a critical factor, and characteristic gene expression was noted in the LcS displaying improved stress tolerance. *In vivo* testing by using human consumption tests confirmed the greater viability of LcS produced by co-culture. In summary, we showed here that the stress tolerance of the probiotic strain changed depending on the method of culture.

When LcS was co-cultured with LL-1, the pH dropped faster than in monoculture, probably owing to lactic acid production by the LL-1. Increased stress tolerance of LcS was also observed in co-culture with *S. thermophilus* YIT 2021, which induced a pH fluctuation similar to that found in the LL-1 co-culture. However, a slower or faster pH decline than in LL-1 co-culture did not significantly improve stress tolerance, suggesting that the period of pH reduction that effectively increases stress tolerance is limited. Moreover, LL-1 co-culture improved stress tolerance in *L. paracasei* but not in *L. helveticus* and *L. gasseri*, which also have the *cfa* gene. We assumed that the degree and timing of the pH reduction required to induce increased stress tolerance varied among the strains. For the application of this strategy to other strains, it will be necessary to elucidate the mechanism of why it works with *L. paracasei* and not with *L. helveticus* or *L. gasseri*. Co-culture improved stress tolerance only in *L. paracasei* in this study, and it cannot be ruled out that this improvement is specific to this bacterial species. Further validations with different combinations of strains are required. Co-culture can also produce metabolites not available in monoculture owing to the inter-strain conversion of molecules. The combination of LcS and LL-1 can produce gamma-aminobutyrate through the degradation of casein protein in milk medium by LcS and the glutamic acid decarboxylase activity of LL-1 (42). The blood-pressure-lowering effect of this beverage has also been confirmed (43). Therefore, co-culture may improve not only probiotic survival but also the production of beneficial metabolites.

Several studies have reported that changes in the ratio of saturated to unsaturated fatty acids and cyclopropane-type fatty acids in the bacterial cell membrane are associated with stress tolerance (44). Increased cyclopropane-type fatty acid contents have been observed in various microorganisms with improved stress tolerance, including *L. paracasei* (45), but their contribution to stress tolerance in lactic acid bacteria has not yet been experimentally proven. By creating a *cfa*-deficient strain, we demonstrated here that the stress tolerance of LcS was not increased in co-culture when the dihydrosterculic fatty acid content was eliminated. The *cfa*-deficient strain itself also showed some increased stress tolerance via co-culture, suggesting the existence of additional mechanisms besides an increased cyclopropane-type fatty acid content.

Gene expression analysis suggested that co-cultured and pH-controlled monocultured LcS have increased energy production through branched-chain amino acid metabolism. In contrast, the conversion of pyruvate to lactate and the synthesis of nucleic acids, peptidoglycans, and fatty acids were reduced, suggesting that energy consumption was suppressed. Culture at a low pH may have altered the energy balance, thus enhancing viability in a harsh environment. Low environmental pH and exposure to bile acids decrease bacterial intracellular pH (46, 47), but increased expression of the F_0_F_1_-ATPase gene may increase proton efflux capacity and contribute to enhanced stress tolerance. These results are consistent with those of previous stress tolerance studies (21). Our gene-set enrichment analysis predicted that the mechanism of stress tolerance is a complex functional change in energy metabolism, lipid metabolism, and protein repair systems. However, the significance of the various functional changes revealed here is yet to be clarified. Further investigations using mutant strains are required.

Human consumption studies are important for evaluating probiotic viability because it is unclear whether simulated gastric acids and bile are comparable to those in the human gastrointestinal tract. We consider that calculation of the viability rates of bacteria through whole-feces collection is the most reliable method, and to our knowledge, this study is the first to use this method. However, the viability rates and CFU densities of LcS were strongly correlated, indicating that CFU density evaluation may also be sufficient. We believe that upper gastrointestinal stress is critical for probiotic survival because we have studied probiotic viability in the terminal ileum and found their recovery to be comparable to that from the feces (48). Therefore, we speculated that individual differences in the viability rate may be due to differences in the upper gastrointestinal tract environment (pH and retention time in the stomach, and composition and concentration of bile). Subjects with less than 1% LcS viability rates when they consumed Beverage A had increased viability rates when they consumed Beverage B. In contrast, those with more than 1% viability rates when they consumed Beverage A had similar viability rates when they consumed Beverage B. We consider that the former subjects had a harsher upper gastrointestinal tract environment with strong antimicrobial activity of the gastric acid and bile, suggesting that the stress-tolerant LcS in Beverage B were more advantageous for survival. In contrast, the latter subjects likely had a less severe upper gastrointestinal environment than the former subjects, so the LcS in both beverages survived to the same degree. However, this hypothesis needs to be confirmed by using a gastrointestinal environment monitoring system like a capsule endoscope. Our *in vitro* validation suggested that the culture conditions had a significant impact on LcS viability, but one limitation of this human study is that the influence of other factors, such as ingredients, cannot be excluded. As it takes time to ensure the safety of cultured bacterial solutions prepared in the laboratory, we used commercialized products in the consumption study. Live probiotic strains have been reported to have greater health benefits than dead strains [e.g., there is a difference in antidiabetic effect (49)], suggesting that highly viable probiotic strains would have greater health benefits. Health effects (e.g., improvement in bowel movements) due to differences in the viability of probiotics will be a topic that should be addressed in the future study. We hope that this research will advance the understanding of probiotics and their impacts on human health.

## MATERIALS AND METHODS

### Bacterial growth and media

The bacterial strains and culture conditions used in this study are listed in Table 1. All strains were cultured in a 10% skim milk medium (Difco Skim Milk, BD, Sparks, MD, USA). For the monoculture of bacterial strains, precultures were inoculated into fresh milk media at 0.5% (vol/vol). For co-culture, individual precultures were inoculated at 0.5% (vol/vol, *L. paracasei*, *L. helveticus*, and *L. gasseri*) or 0.01% (vol/vol, LL-1 and *S. thermophilus*). Both monocultures and co-cultures were grown for 48 h at 37°C.

**TABLE 1:** Bacterial strains and culture conditions used in this study

Strain name	Abbreviation	Note	Other organization registration no.	Source	Culture conditions
*Lacticaseibacillus paracasei* Shirota YIT 9029	LcS		FERM P-5852; FERM BP-1366	Lab collection	37°C for 2 days
*Lactococcus lactis* subsp. *lactis* YIT 2027	LL-1		FERM P-16074; FERM BP-6224	Lab collection	30°C for 1 day
*Streptococcus thermophilus* YIT 2001			FERM P-1767; FERM P-11891; FERM P-18189; FERM BP-7538	Lab collection	37°C for 1 day
*Streptococcus thermophilus* YIT 2021			FERM P-15933; FERM BP-7537	Lab collection	37°C for 1 day
*Streptococcus thermophilus* YIT 2037		Type strain	ATCC 19258; NCDO 573; NCIB 8510; DSM 20617	Lab collection	37°C for 1 day
*Lactobacillus helveticus* YIT 0083		Type strain	ATCC 15009; JCM 1120; DSM 20075; IFO 15019; NCFB 2712; NRIC 1545	Lab collection	37°C for 2 days
*Lactobacillus gasseri* YIT 0168			JCM 5813; FERM P-6262; FERM BP-7536	Lab collection	37°C for 2 days
*Lacticaseibacillus paracasei* YIT 0180			ATCC 334	Lab collection	37°C for 2 days
*Lacticaseibacillus paracasei* YIT 0209		Type strain	NCDO 151; ATCC 25302; JCM 8130; DSM 5622; NCIMB 700151	Lab collection	37°C for 2 days
LcS Δ*cfa*	Δ*cfa*			This study	37°C for 2 days

### CFU, pH, and DO measurement

To determine the number of viable bacteria, serial dilutions of the cultures were inoculated onto plates of lactitol-LBS-vancomycin (LLV) agar (50) (a selective agar medium for *Lacticaseibacillus*) or MRS agar plates with 0.5 µg/mL tetracycline in the case of *L. gasseri* and *L. helveticus*. After 3 days of incubation of the cultures at 37°C, the CFUs were counted. pH was measured with a pH meter (D-71, Horiba, Kyoto, Japan), and DO was measured with a DO meter (HQ30d, Hach Company, CO, USA).

### pH-controlled culture

pH-controlled monoculture and its comparison with monoculture and co-culture were conducted by using a 250-mL jar fermenter (Bio Jr.8, Able Corp, Tokyo, Japan), which can control temperature, pH, DO, and stirring conditions. For all cultures, 100 mL of 10% skim milk medium was used. The temperature was set at 37°C with agitation at 75 rpm for 48 h. During pH-controlled culture, 2N lactic acid was automatically added according to a programmed protocol, by using the accompanying software. The preculture conditions and inoculation conditions were as described above.

### Simulated gastrointestinal stress

Lactic acid bacteria cultures (2 mL) were added to 78 mL of simulated gastric acid containing 10 g of Proteose Peptone No. 3 (BD), 3 g of mucin (Wako, Osaka, Japan), 10 g of NaCl (Manac, Tokyo, Japan), 6 g of NaHCO_3_ (Wako), 2 g of KH_2_PO_4_ (Wako), and a small amount of HCl (Wako) for pH adjustment per liter. Bacterial cultures were incubated in pH-4.0 simulated gastric acid with agitation at 75 rpm for 1 h, then 0.1 M NaHCO_3_ was added to raise the pH to 4.4 and simulated bile containing 0.3% oxgall solution (BD) was added. This was followed by incubation for 5 min at 37°C. Then, 0.1 mL of the reaction fluid obtained after each incubation step was harvested in 0.9 mL of PBS and plate counting was performed as described above to determine the number of surviving LcS.

### Total RNA extraction

For total RNA extraction, 20 mL of RNAprotect Bacteria Reagent (Qiagen, Hilden, Germany) was added to 10 mL of bacterial culture, and conditioning of cell pellets was done according to the Qiagen manual. The cell pellets were suspended in 5 mL of 50 mM glycine buffer (pH 3.0) with a final concentration of 10 mg/mL trypsin (Affymetrix, Santa Clara, CA, USA) and incubated at 37°C for 10 min to degrade casein. Cells were harvested by centrifugation for 10 min at 5,000 × *g* and suspended in RNA extraction buffer containing 343 µL of RLT (Qiagen), 100 µL of Tris-EDTA (TE), 7 µL of 2 M Dithiothreitol (DTT), and 0.3 g of φ 0.1 mm glass beads. After the bacterial cells had been disrupted by using a Shake Master (Biomedical Science, Tokyo, Japan), 500 µL of water-saturated phenol was added, and the mixture was allowed to stand at 60°C for 10 min. Then, 100 µL of chloroform/isoamyl alcohol (24:1) was added, the mixture was centrifuged at 13,000 × *g* for 5 min, and isopropanol precipitation and ethanol precipitation were performed. The RNA integrity of all samples was confirmed with an Agilent 2100 Bioanalyzer (Agilent Technologies, Santa Clara, CA, USA).

### Microarray analysis

A custom oligonucleotide spotted microarray (ID: 074275) based on the genome sequence of LcS was designed, produced, and validated by using the eArray system (Agilent Technologies). Cyanine-3-labeled cRNA was obtained from 100 ng of extracted total RNA by using a Low Input Quick Amp WT Labeling Kit (Agilent Technologies). Hybridization was performed on custom microarrays at 65°C for 17 h by using the prepared cRNA. The slides were then washed, and images of the microarrays were acquired by using an Agilent G4900DA SureScan Scanner (Agilent Technologies). The signal data for each spot were subsequently quantified by using Feature Extraction software (Agilent Technologies). The limma package (v.3.36.5) (51) of the statistical analysis software R (v4.2.1) was used for background correction, quantile normalization of the probe signal data, and identification of differentially expressed genes. The NCBI’s Conserved Domain Search was utilized for the COG classification of genes. Enrichment analysis of COGs was performed by using the clusterProfiler package (v4.4.4) (52).

### Membrane fatty acid composition analysis

To remove the milk components from the culture medium, 240 mL of Tris-EDTA solution (pH 9.5) was added to 80 mL of the bacterial culture, and the mixture was centrifuged at 5,000 × *g* for 20 min. The bacterial precipitates were then suspended in 30 mM EDTA (pH 12) and reacted at room temperature for 60 min to solubilize the milk components. The mixture was centrifuged again at 5,000 × *g* for 20 min to remove the supernatant, and the bacterial pellets were washed three times with saline solution. Fatty acid extraction and compositional measurements were performed by TechnoSuruga Laboratory Co. Ltd (Shizuoka, Japan).

### Construction of the *cfa*-deficient strain

The *cfa*-deficient strain was constructed by deleting the *cfa* gene, as previously reported (53). The primer pairs used to amplify fragments containing the 5′- and 3′-terminal ends of the *cfa* gene are listed in Table 2. Gene-disrupting plasmid was introduced into LcS, and the *cfa*-deficient strain was constructed by using a stepwise double-crossover method.

**TABLE 2:** Primers used in this study

Primer name	Sequence (5′–3′)	Application
InF_pYSSE3_F	ACCTGCAGGCATGCAAGCTT	Amplified pYSSE3 for In-Fusion cloning of upstream and downstream regions of the *cfa*
InF_pYSSE3_R	CGGGTACCGAGCTCGAATTC	
cfa_A_F	AGCCTGAGCAGTGACATTTG	Amplified upstream region of the *cfa*
cfa_A_R	CTTAATTATCGCGGTTGACCGATGGCCTCCGTTTTGAGAA	
cfa_B_F	TTCTCAAAACGGAGGCCATCGGTCAACCGCGATAATTAAG	Amplified downstream region of the *cfa*
cfa_B_R	TGAGTGGGTGGCTCATTTCT	
cfa_Inf_F	CGAGCTCGGTACCCGAGCCTGAGCAGTGACATTT	Amplified flanking fragments upstream and downstream of *cfa* for In-Fusion cloning,
cfa_Inf_R	TGCATGCCTGCAGGTTGAGTGGGTGGCTCATTTC	

### Human trials

Before enrollment, participants provided written informed consent after being fully informed of the study’s objectives, methods, potential risks, and benefits. Twenty-four subjects were recruited and allocated to two groups (Groups I and II) to participate in a crossover trial evaluating the consumption of two test beverages: “Beverage A,” which comprised an LcS-fermented milk product, and “Beverage B,” which comprised an LcS and LL-1 co-fermented milk product. The mean age of the subjects was 36.9 years (SD, 7.6 years) and all were male. Following the study onset, participants were instructed to abstain from consuming yogurt, fermented milk beverages, oligosaccharide-added foods, and xylitol-added foods, in addition to products containing LcS. After a 6-day pre-consumption observation period, subjects consumed one bottle of Beverage A (Group I) or Beverage B (Group II), and all whole-stool samples expelled during the subsequent 4-day period were collected (Test Phase 1). Following a 3-day washout period, the subjects consumed one bottle of Beverage B (Group I) or Beverage A (Group II) and all whole-stool samples were again collected during the subsequent 4 days (Test Phase 2). To confirm that LcS was not detectable in the feces before consumption of the test beverage, feces were collected once during the pre-consumption observation period and once in the washout period. Two individuals were excluded from the analysis because of sample collection failure. Fecal dilutions were plated onto LLV agar, and the CFUs per gram of feces were determined. The numbers of viable LcS in the feces and test beverages were calculated by multiplying the CFU densities by the weight or volume, respectively. The viability rate of LcS was calculated by dividing the sum of the number of viable LcS recovered from all the feces by the number of viable LcS in the product consumed.

## Data Availability

The raw microarray data and the differential expression analysis results obtained by using limma are available from the Zenodo database (https://zenodo.org/records/8260018).

## SUPPLEMENTAL MATERIAL

Supplementary figure legends aem.01502-23-s0001.doc Supplementary Figure Legends for Fig. S1 to Fig. S7

Fig. S1 aem.01502-23-s0002.pdf Changes in LcS CFU, LL-1 CFU, and medium pH during co-culture.

Fig. S2 aem.01502-23-s0003.pdf Stress tolerance and culture properties of anaerobically cultured LcS.

Fig. S3 aem.01502-23-s0004.pdf Dissimilarity of pH changes among the cultures in Fig. 3B.

Fig. S4 aem.01502-23-s0005.pdf Cell-membrane fatty acid composition of each culture and strain.

Fig. S5 aem.01502-23-s0006.pdf Gene expression analysis and morphological observation of LcS.

Fig. S6 aem.01502-23-s0007.pdf Weight of fecal material collected, and relationship between viability rate and CFU density of LcS.

Fig. S7 aem.01502-23-s0008.pdf Effect of *in vitro* gastrointestinal stress on the number of LcS cells and the percentage of LcS cell counts in feces after consumption of beverages.

ASM does not own the copyrights to Supplemental Material that may be linked to, or accessed through, an article. The authors have granted ASM a non-exclusive, world-wide license to publish the Supplemental Material files. Please contact the corresponding author directly for reuse.

## References

[1] Lilly DM, Stillwell RH. 1965. Probiotics: growth-promoting factors produced by microorganisms. Science 147:747–748. doi:10.1126/science.147.3659.747 14242024

[2] Afrc RF. 1989. Probiotics in man and animals. J Appl Bacteriol 66:365–378. doi:10.1111/j.1365-2672.1989.tb05105.x 2666378

[3] Health and nutritional properties of Probiotics in food including powder milk with live lactic acid bacteria: Report of a joint FAO WHO expert consultation on evaluation of health and nutritional properties of Probiotics in food including powder milk with live lactic acid bacteria. 2001. American Córdoba Park Hotel, Córdoba, Argentina

[4] Nagata S, Asahara T, Wang C, Suyama Y, Chonan O, Takano K, Daibou M, Takahashi T, Nomoto K, Yamashiro Y. 2016. The effectiveness of Lactobacillus beverages in controlling infections among the residents of an aged care facility: a randomized placebo-controlled double-blind trial. Ann Nutr Metab 68:51–59. doi:10.1159/000442305 26599038

[5] Koebnick C, Wagner I, Leitzmann P, Stern U, Zunft HJF. 2003. Probiotic beverage containing Lactobacillus casei shirota improves gastrointestinal symptoms in patients with chronic constipation. Can J Gastroenterol 17:655–659. doi:10.1155/2003/654907 14631461

[6] Aoki T, Asahara T, Matsumoto K, Takada T, Chonan O, Nakamori K, Nonaka C, Yamaji I, Hisamoto T, Sato M, Matsuda T, Nomoto K. 2014. Effects of the continuous intake of a milk drink containing Lactobacillus casei strain shirota on abdominal symptoms, fecal microbiota, and metabolites in gastrectomized subjects. Scand J Gastroenterol 49:552–563. doi:10.3109/00365521.2013.848469 24621348

[7] Takeda K, Suzuki T, Shimada S-I, Shida K, Nanno M, Okumura K. 2006. Interleukin-12 is involved in the enhancement of human natural killer cell activity by Lactobacillus casei shirota. Clin Exp Immunol 146:109–115. doi:10.1111/j.1365-2249.2006.03165.x 16968405 PMC1809741

[8] Shida K, Sato T, Iizuka R, Hoshi R, Watanabe O, Igarashi T, Miyazaki K, Nanno M, Ishikawa F. 2017. Daily intake of fermented milk with Lactobacillus casei strain shirota reduces the incidence and duration of upper respiratory tract infections in healthy middle-aged office workers. Eur J Nutr 56:45–53. doi:10.1007/s00394-015-1056-1 26419583 PMC5290054

[9] Takada M, Nishida K, Gondo Y, Kikuchi-Hayakawa H, Ishikawa H, Suda K, Kawai M, Hoshi R, Kuwano Y, Miyazaki K, Rokutan K. 2017. Beneficial effects of Lactobacillus casei strain shirota on academic stress-induced sleep disturbance in healthy adults: a double-blind, randomised, placebo-controlled trial. Benef Microbes 8:153–162. doi:10.3920/BM2016.0150 28443383

[10] Sarkar S. 2018. Whether viable and dead probiotic are equally efficacious?. NFS 48:285–300. doi:10.1108/NFS-07-2017-0151

[11] Adams CA. 2010. The probiotic paradox: live and dead cells are biological response modifiers. Nutr Res Rev 23:37–46. doi:10.1017/S0954422410000090 20403231

[12] Lahtinen SJ. 2012. Probiotic viability - does it matter? Microb Ecol Health Dis 23. doi:10.3402/mehd.v23i0.18567 PMC3747757 23990833

[13] Plaza-Diaz J, Ruiz-Ojeda FJ, Gil-Campos M, Gil A. 2019. Mechanisms of action of probiotics. Adv Nutr 10:S49–S66. doi:10.1093/advances/nmy063 30721959 PMC6363529

[14] Castro-López C, Romero-Luna HE, García HS, Vallejo-Cordoba B, González-Córdova AF, Hernández-Mendoza A. 2023. Key stress response mechanisms of probiotics during their journey through the digestive system: a review. Probiotics Antimicro Prot 15:1250–1270. doi:10.1007/s12602-022-09981-x 36001271

[15] Corcoran BM, Stanton C, Fitzgerald G, Ross RP. 2008. Life under stress: the probiotic stress response and how it may be manipulated. Curr Pharm Des 14:1382–1399. doi:10.2174/138161208784480225 18537661

[16] Giraffa G. 2012. Selection and design of lactic acid bacteria probiotic cultures. Eng Life Sci 12:391–398. doi:10.1002/elsc.201100118

[17] Lacroix C, Yildirim S. 2007. Fermentation technologies for the production of probiotics with high viability and functionality. Curr Opin Biotechnol 18:176–183. doi:10.1016/j.copbio.2007.02.002 17336510

[18] Upadrasta A, Stanton C, Hill C, Fitzgerald GF, Ross RP. 2011. Improving the stress tolerance of probiotic cultures: recent trends and future directions, p 395–438. In Tsakalidou E, K Papadimitriou (ed), Stress responses of lactic acid bacteria. Springer US, Boston, MA.

[19] Corcoran BM, Stanton C, Fitzgerald GF, Ross RP. 2007. Growth of Probiotic Lactobacilli in the presence of oleic acid enhances subsequent survival in gastric juice. Microbiology (Reading) 153:291–299. doi:10.1099/mic.0.28966-0 17185558

[20] Gaucher F, Bonnassie S, Rabah H, Marchand P, Blanc P, Jeantet R, Jan G. 2019. Review: adaptation of beneficial propionibacteria, Lactobacilli, and bifidobacteria improves tolerance toward technological and digestive stresses. Front Microbiol 10:841. doi:10.3389/fmicb.2019.00841 31068918 PMC6491719

[21] Papadimitriou K, Alegría Á, Bron PA, de Angelis M, Gobbetti M, Kleerebezem M, Lemos JA, Linares DM, Ross P, Stanton C, Turroni F, van Sinderen D, Varmanen P, Ventura M, Zúñiga M, Tsakalidou E, Kok J. 2016. Stress physiology of lactic acid bacteria. Microbiol Mol Biol Rev 80:837–890. doi:10.1128/MMBR.00076-15 27466284 PMC4981675

[22] Burgain J, Gaiani C, Linder M, Scher J. 2011. Encapsulation of probiotic living cells: from laboratory scale to industrial applications. J Food Eng 104:467–483. doi:10.1016/j.jfoodeng.2010.12.031

[23] Chassaing B, Koren O, Goodrich JK, Poole AC, Srinivasan S, Ley RE, Gewirtz AT. 2015. Dietary emulsifiers impact the mouse gut microbiota promoting colitis and metabolic syndrome. 7541. Nature 519:92–96. doi:10.1038/nature14232 25731162 PMC4910713

[24] Jin G, Tang Q, Ma J, Liu X, Zhou B, Sun Y, Pang X, Guo Z, Xie R, Liu T, Wang B, Cao H, Zhou H. 2021. Maternal emulsifier P80 intake induces gut dysbiosis in offspring and increases their susceptibility to colitis in adulthood. mSystems 6:e01337-20. doi:10.1128/mSystems.01337-20 33727402 PMC8547008

[25] Fiocco D, Longo A, Arena MP, Russo P, Spano G, Capozzi V. 2020. How probiotics face food stress: they get by with a little help. Crit Rev Food Sci Nutr 60:1552–1580. doi:10.1080/10408398.2019.1580673 30880406

[26] Reque PM, Brandelli A. 2021. Encapsulation of probiotics and nutraceuticals: applications in functional food industry. Trends Food Sci 114:1–10. doi:10.1016/j.tifs.2021.05.022

[27] Teuber M. 2015. Lactococcus, p 1–21. In In bergey’s manual of systematics of archaea and bacteria. John Wiley & Sons, Ltd.

[28] Sohlenkamp C, Geiger O. 2016. Bacterial membrane lipids: diversity in structures and pathways. FEMS Microbiol Rev 40:133–159. doi:10.1093/femsre/fuv008 25862689

[29] Brown JL, Ross T, McMeekin TA, Nichols PD. 1997. Acid habituation of Escherichia coli and the potential role of cyclopropane fatty acids in low pH tolerance. Int J Food Microbiol 37:163–173. doi:10.1016/s0168-1605(97)00068-8 9310851

[30] Grandvalet C, Assad-García JS, Chu-Ky S, Tollot M, Guzzo J, Gresti J, Tourdot-Maréchal R. 2008. Changes in membrane lipid composition in ethanol- and acid-adapted oenococcus oeni cells: characterization of the cfa gene by heterologous complementation. Microbiology (Reading) 154:2611–2619. doi:10.1099/mic.0.2007/016238-0 18757795

[31] Chang Y-Y, Cronan JE. 1999. Membrane cyclopropane fatty acid content is a major factor in acid resistance of Escherichia coli. Mol Microbiol 33:249–259. doi:10.1046/j.1365-2958.1999.01456.x 10411742

[32] Lebeer S, Claes IJJ, Verhoeven TLA, Shen C, Lambrichts I, Ceuppens JL, Vanderleyden J, De Keersmaecker SCJ. 2008. Impact of luxS and suppressor mutations on the gastrointestinal transit of Lactobacillus rhamnosus GG. Appl Environ Microbiol 74:4711–4718. doi:10.1128/AEM.00133-08 18539797 PMC2519330

[33] Whitehead K, Versalovic J, Roos S, Britton RA. 2008. Genomic and genetic characterization of the bile stress response of probiotic Lactobacillus reuteri ATCC 55730. Appl Environ Microbiol 74:1812–1819. doi:10.1128/AEM.02259-07 18245259 PMC2268311

[34] Arena MP, Capozzi V, Longo A, Russo P, Weidmann S, Rieu A, Guzzo J, Spano G, Fiocco D. 2019. The phenotypic analysis of Lactobacillus plantarum shsp mutants reveals a potential role for hsp1 in cryotolerance. Front Microbiol 10:838. doi:10.3389/fmicb.2019.00838 31114549 PMC6503756

[35] Wu C, Zhang J, Du G, Chen J. 2013. Aspartate protects Lactobacillus casei against acid stress. Appl Microbiol Biotechnol 97:4083–4093. doi:10.1007/s00253-012-4647-2 23292549

[36] Tatusov RL, Koonin EV, Lipman DJ. 1997. A genomic perspective on protein families. Science 278:631–637. doi:10.1126/science.278.5338.631 9381173

[37] Futai M, Noumi T, Maeda M. 1989. ATP synthase (H+-ATPase): results by combined biochemical and molecular biological approaches. Annu Rev Biochem 58:111–136. doi:10.1146/annurev.bi.58.070189.000551 2528322

[38] Smokvina T, Wels M, Polka J, Chervaux C, Brisse S, Boekhorst J, van Hylckama Vlieg JET, Siezen RJ. 2013. Lactobacillus paracasei comparative genomics: towards species pan-genome definition and exploitation of diversity. PLoS One 8:e68731. doi:10.1371/journal.pone.0068731 23894338 PMC3716772

[39] Holbrook JJ, Liljas A, Steindel SJ, Rossmann MG. 1975. 4 lactate dehydrogenase, p 191–292. In Boyer PD (ed), The enzymes. Academic Press.

[40] Hove-Jensen B. 1988. Mutation in the phosphoribosylpyrophosphate synthetase gene (prs) that results in simultaneous requirements for purine and pyrimidine nucleosides, nicotinamide nucleotide, histidine, and tryptophan in Escherichia coli. J Bacteriol 170:1148–1152. doi:10.1128/jb.170.3.1148-1152.1988 2449419 PMC210885

[41] Benson TE, Walsh CT, Hogle JM. 1996. The structure of the substrate-free form of MurB, an essential enzyme for the synthesis of bacterial cell walls. Structure 4:47–54. doi:10.1016/s0969-2126(96)00008-1 8805513

[42] 2010. Biotechnology in functional foods and nutraceuticals. CRC Press, Boca Raton.

[43] Inoue K, Shirai T, Ochiai H, Kasao M, Hayakawa K, Kimura M, Sansawa H. 2003. Blood-pressure-lowering effect of a novel fermented milk containing γ-aminobutyric acid (GABA) in mild hypertensives. Eur J Clin Nutr 57:490–495. doi:10.1038/sj.ejcn.1601555 12627188

[44] Fozo EM, Kajfasz JK, Quivey RG. 2004. Low pH-induced membrane fatty acid alterations in oral bacteria. FEMS Microbiol Lett 238:291–295. doi:10.1016/j.femsle.2004.07.047 15358413

[45] Broadbent JR, Larsen RL, Deibel V, Steele JL. 2010. Physiological and transcriptional response of Lactobacillus casei ATCC 334 to acid stress . J Bacteriol 192:2445–2458. doi:10.1128/JB.01618-09 20207759 PMC2863488

[46] Siegumfeldt H, Björn Rechinger K, Jakobsen M. 2000. Dynamic changes of intracellular pH in individual lactic acid bacterium cells in response to a rapid drop in extracellular pH. Appl Environ Microbiol 66:2330–2335. doi:10.1128/AEM.66.6.2330-2335.2000 10831407 PMC110524

[47] Kurdi P, Kawanishi K, Mizutani K, Yokota A. 2006. Mechanism of growth inhibition by free bile acids in Lactobacilli and bifidobacteria. J Bacteriol 188:1979–1986. doi:10.1128/JB.188.5.1979-1986.2006 16484210 PMC1426545

[48] Takada T, Chinda D, Mikami T, Shimizu K, Oana K, Hayamizu S, Miyazawa K, Arai T, Katto M, Nagara Y, Makino H, Kushiro A, Oishi K, Fukuda S. 2020. Dynamic analysis of human small intestinal microbiota after an ingestion of fermented milk by small-intestinal fluid perfusion using an endoscopic retrograde bowel insertion technique. Gut Microbes 11:1662–1676. doi:10.1080/19490976.2020.1766942 32552401 PMC7524281

[49] Li X, Xu Q, Jiang T, Fang S, Wang G, Zhao J, Zhang H, Chen W. 2016. A comparative study of the antidiabetic effects exerted by live and dead multi-strain probiotics in the type 2 diabetes model of mice. Food Funct 7:4851–4860. doi:10.1039/c6fo01147k 27812581

[50] Yuki N, Watanabe K, Mike A, Tagami Y, Tanaka R, Ohwaki M, Morotomi M. 1999. Survival of a probiotic, Lactobacillus casei strain shirota, in the gastrointestinal tract: selective isolation from faeces and identification using monoclonal antibodies. Int J Food Microbiol 48:51–57. doi:10.1016/s0168-1605(99)00029-x 10375134

[51] Ritchie ME, Phipson B, Wu D, Hu Y, Law CW, Shi W, Smyth GK. 2015. Limma powers differential expression analyses for RNA-sequencing and microarray studies. Nucleic Acids Res 43:e47. doi:10.1093/nar/gkv007 25605792 PMC4402510

[52] Yu G, Wang L-G, Han Y, He Q-Y. 2012. ClusterProfiler: an R package for comparing biological themes among gene clusters. OMICS 16:284–287. doi:10.1089/omi.2011.0118 22455463 PMC3339379

[53] Serata M, Iino T, Yasuda E, Sako T. 2012. Roles of thioredoxin and thioredoxin reductase in the resistance to oxidative stress in Lactobacillus casei. Microbiology (Reading) 158:953–962. doi:10.1099/mic.0.053942-0 22301908

