# Supplementary material for: Manipulating the growth environment through co-culture to enhance stress tolerance and viability of probiotic strains in the gastrointestinal tract
Source: Appl Environ Microbiol. 2023 Nov 29;89(12):e01502-23. doi: 10.1128/aem.01502-23 (PMC10734474; doi:10.1128/aem.01502-23)
Supplement: Supplementary figure legends — for Fig. S1 to Fig. S7 [file aem.01502-23-s0001.doc]

**Supplementary Figure Legends**

**Figure S1.**

Changes in LcS CFU, LL-1 CFU, and medium pH during co-culture

**Figure S2.**

Stress tolerance and culture properties of anaerobically cultured LcS. (A) Log10 (CFU/mL) of LcS after pH-3.0 gastric acid challenge in monoculture and anaerobic culture (mean ± SD, *n* = 3). For the gastric acid challenge, bacterial cultures were incubated in pH-3.0 simulated gastric acid at 37 °C with agitation at 75 rpm for 1 h. Statistical significance was determined by using a two-tailed Welch’s *t*-test (NS, not significant). (B) Log10 (CFU/mL), pH, and DO changes during monoculture, co-culture, and anaerobic culture (mean ± SD, *n* = 3).

**Figure S3.**

Dissimilarity of pH changes among the cultures in Fig. 3B. Dissimilarity was calculated by using the TSclust package in R. The color key indicates the Euclidean distance dissimilarity. The name of each tile indicates the following culture: LcS, LcS monoculture; ST2001, *S. thermophilus* YIT 2001 co-culture; ST2021, *S. thermophilus* YIT 2021 co-culture; ST2037, *S. thermophilus* YIT 2037 co-culture; LL-1, LL-1 co-culture. The number after the underscore indicates the replicate of each culture.

**Figure S4.**

Cell-membrane fatty acid composition of each culture and strain. (A) Comparison between monoculture and pH-controlled monoculture (mean ± SD, *n* = 3, Student’s *t*-test, **, *P* < 0.01; *, *P* < 0.05; NS, not significant). (B) Comparison between wild-type and *cfa*-deficient strains in monoculture (*n* = 1).

**Figure S5.**

Gene expression analysis and morphological observation of LcS. (A) Expression of genes involved in lysine biosynthesis via the diaminopimelate pathway (*dapA*, *dapB*, *dapE*, and *lysA*) in each culture. (B) Expression of *cfa* in each culture. (C) Expression of *prsA* and *murB* in each culture. (A to C) Statistically significant differences compared with monoculture were analyzed by using the limma package (*, Benjamini–Hochberg adjusted *P* value < 0.05; NS, not significant). (D) Expression of genes in the COG M category. (E) Electron micrographs of cross-sections and lengths of each region in monoculture and pH-controlled monoculture. Bars in the electron micrographs indicate 100 nm. Length comparisons were performed with *n* = 20 (mean ± SD, Student’s *t*-test. NS, not significant).

**Figure S6.**

Weight of fecal material collected, and relationship between viability rate and CFU density of LcS. (A) Weight of collected feces (*n* = 202, mean = 141 g, SD = 79.8 g, min = 4 g, max = 368 g). (B) Scatter plot of LcS CFU concentration versus viability rate. CFU concentrations represent the highest values during the test phase. Spearman’s correlation coefficient was 0.938 (*n* = 44).

**Figure S7.**

Effect of *in vitro* gastrointestinal stress on the number of LcS cells and the percentage of LcS cell counts in feces after consumption of beverages. (A) DAPI stained image of cultured LcS before the simulated gastrointestinal stress. (B) DAPI stained image of cultured LcS after the simulated gastrointestinal stress. (C) Log10 (Cells/mL) of LcS before and after the simulated gastrointestinal stress (mean ± SD, n= 9 or 10). (D) Percentage of counts of LcS stained with specific fluorescent antibodies, including viable and non-viable, in recovered feces relative to test beverages (*n* = 22). (C to D) Statistical significances were determined by using two-tailed Welch’s *t*-test (NS, not significant).
