# Supplementary figures and images for: Manipulating the growth environment through co-culture to enhance stress tolerance and viability of probiotic strains in the gastrointestinal tract
Source: Appl Environ Microbiol. 2023 Nov 29;89(12):e01502-23. doi: 10.1128/aem.01502-23 (PMC10734474; doi:10.1128/aem.01502-23)

**Figure S1**

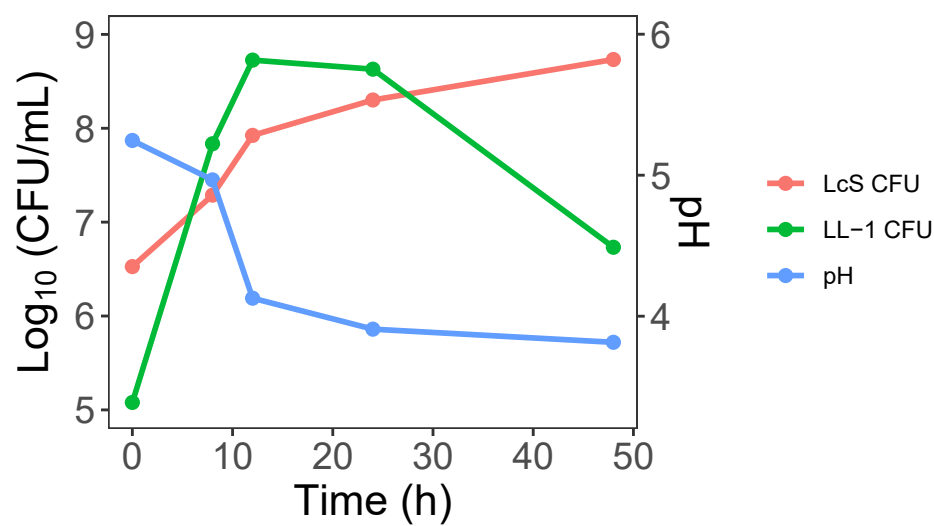

Supplement: Fig. S1 — Changes in LcS CFU, LL-1 CFU, and medium pH during co-culture. [file aem.01502-23-s0002.pdf]

Figure S2

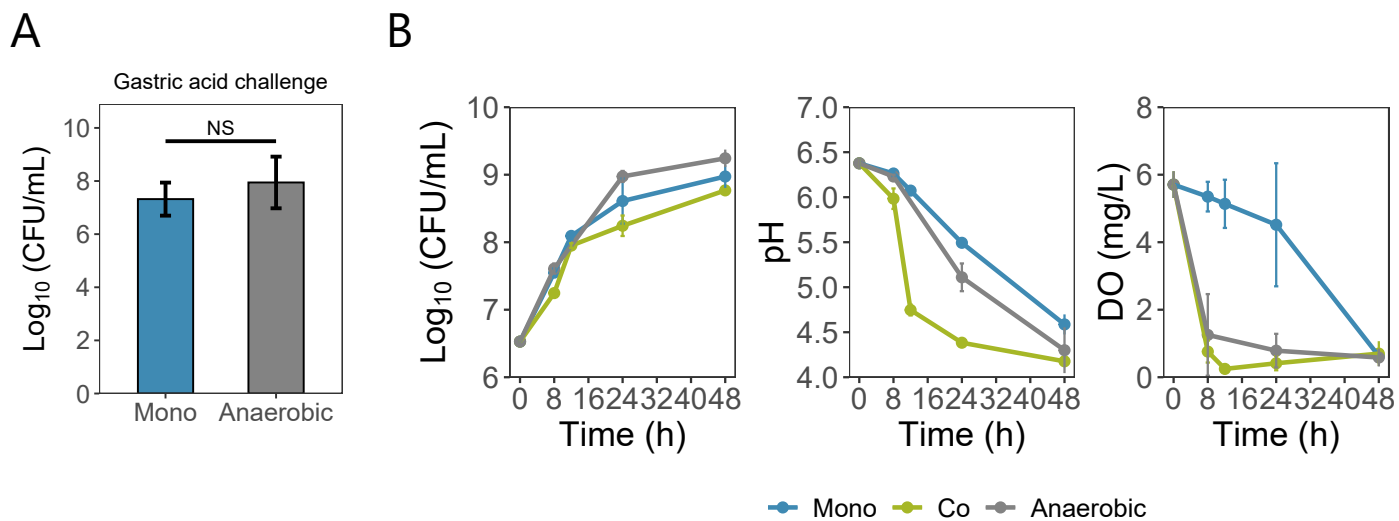

Supplement: Fig. S2 — Stress tolerance and culture properties of anaerobically cultured LcS. [file aem.01502-23-s0003.pdf]

Figure S3

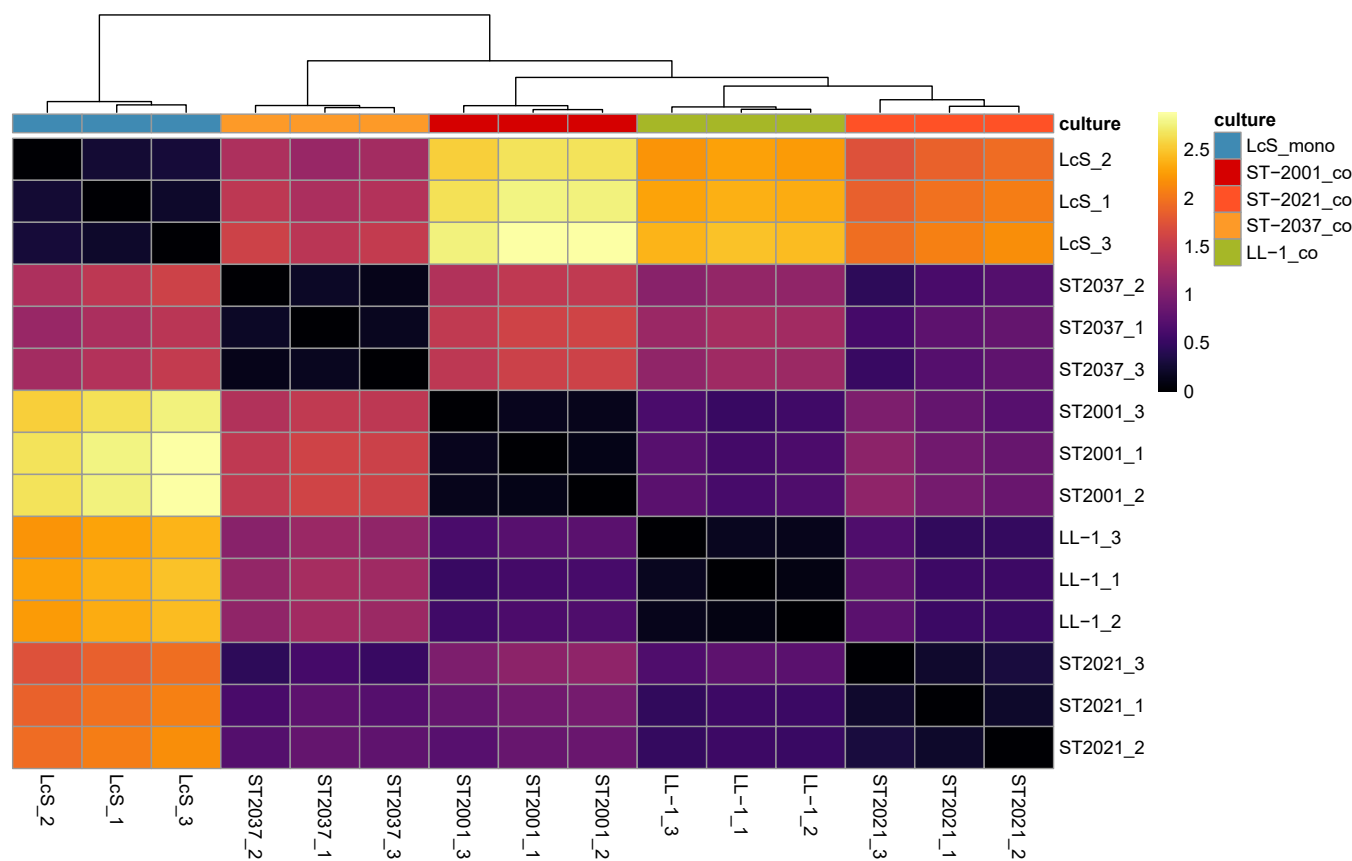

Supplement: Fig. S3 — Dissimilarity of pH changes among the cultures in Fig. 3B. [file aem.01502-23-s0004.pdf]

Figure S4

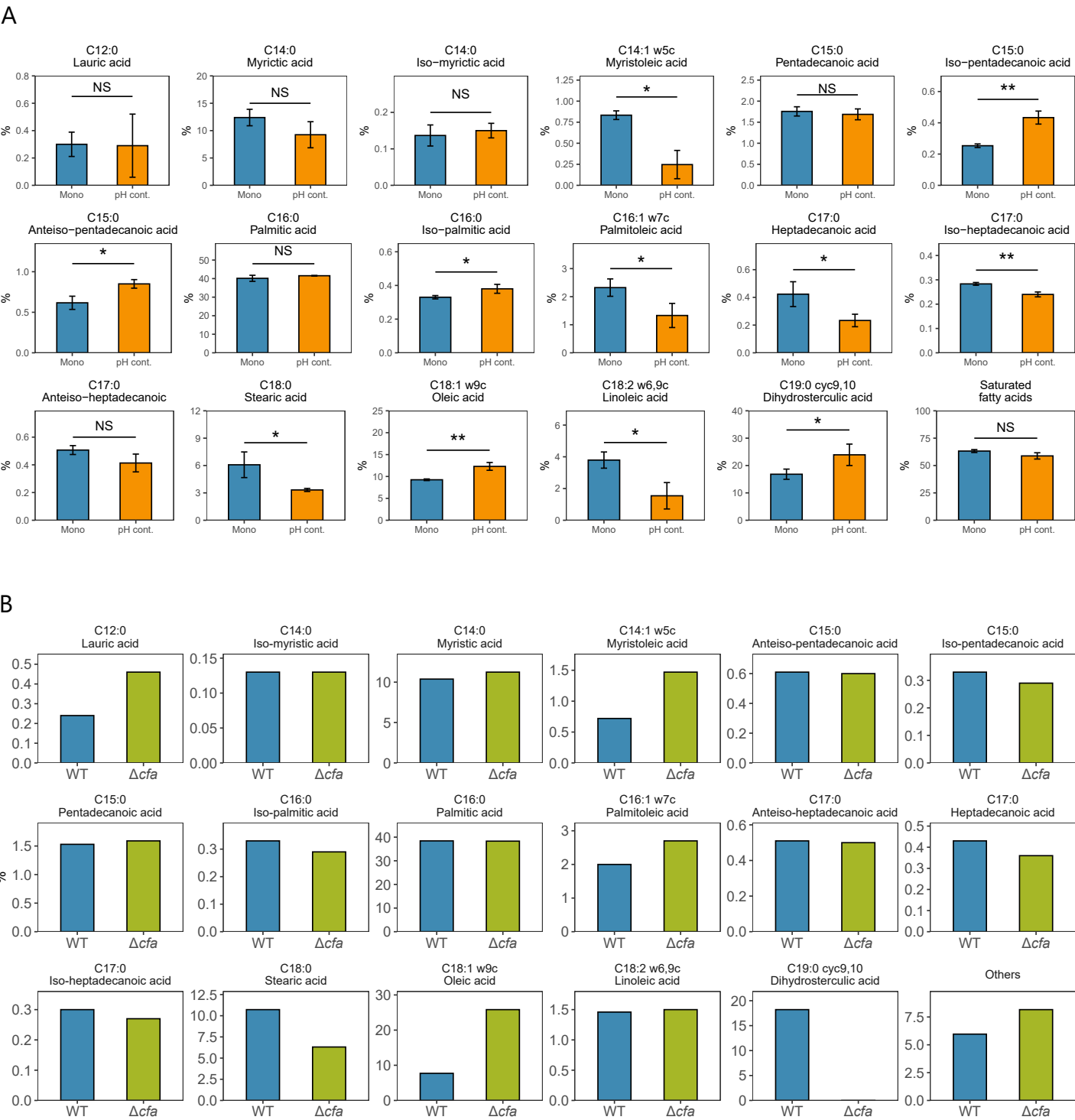

Supplement: Fig. S4 — Cell-membrane fatty acid composition of each culture and strain. [file aem.01502-23-s0005.pdf]

Figure S5

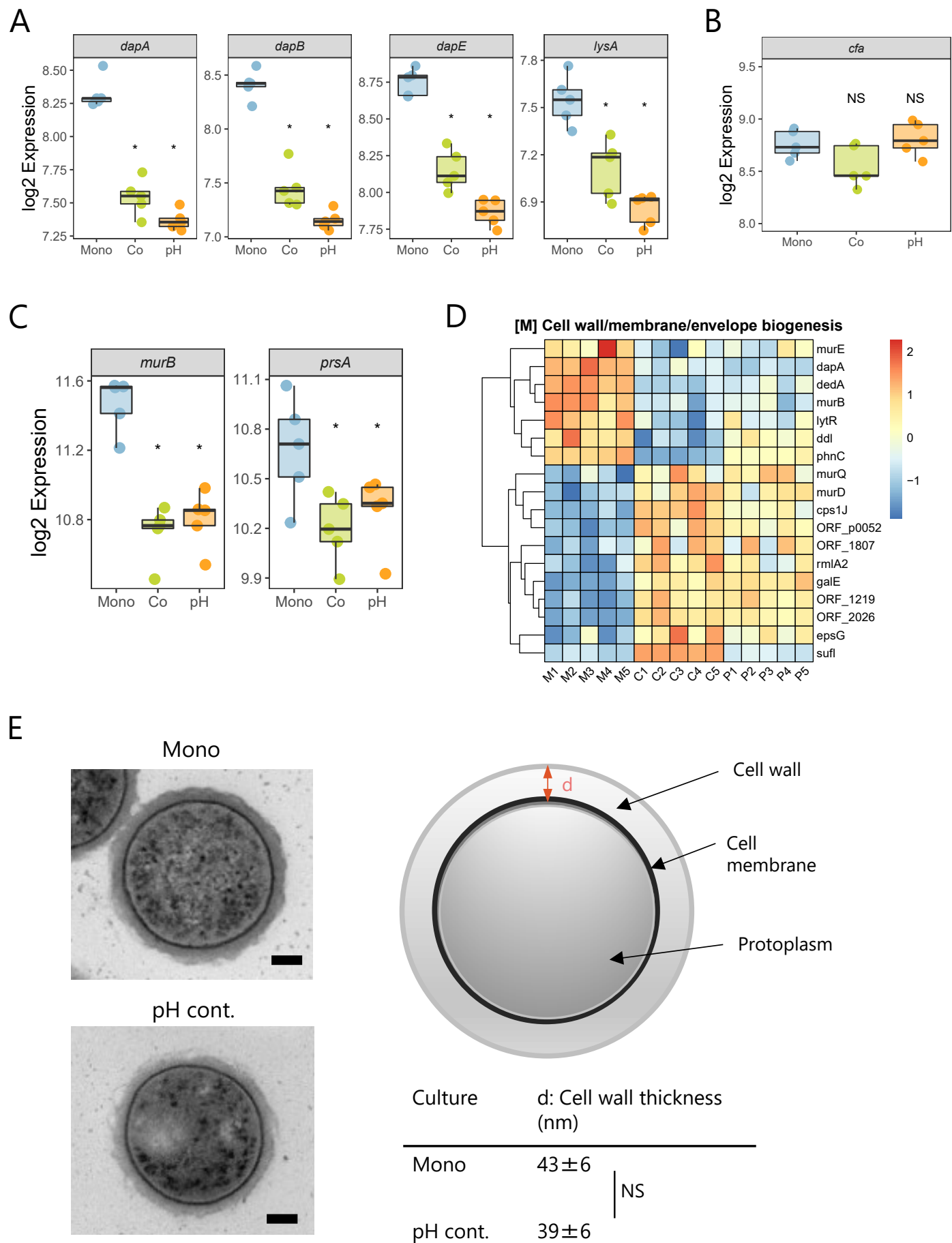

Supplement: Fig. S5 — Gene expression analysis and morphological observation of LcS. [file aem.01502-23-s0006.pdf]

**Figure S6**

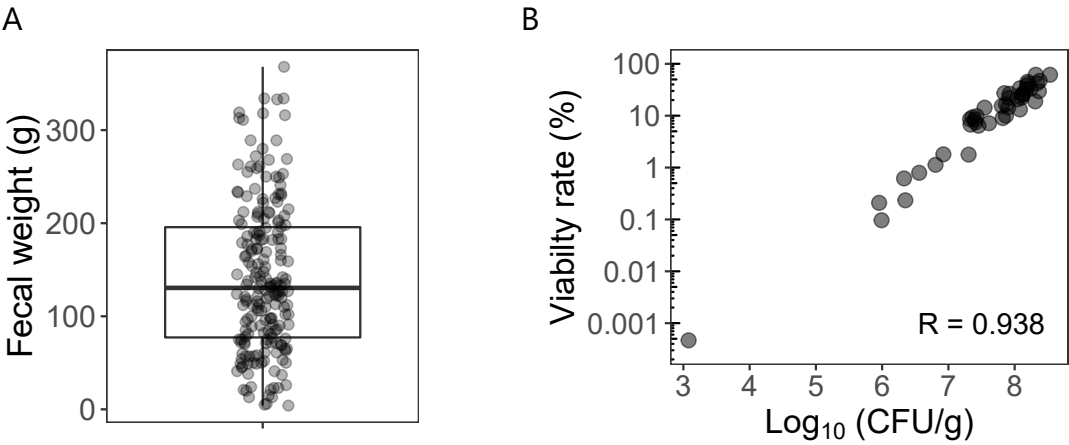

Supplement: Fig. S6 — Weight of fecal material collected, and relationship between viability rate and CFU density of LcS. [file aem.01502-23-s0007.pdf]

**Figure S7**

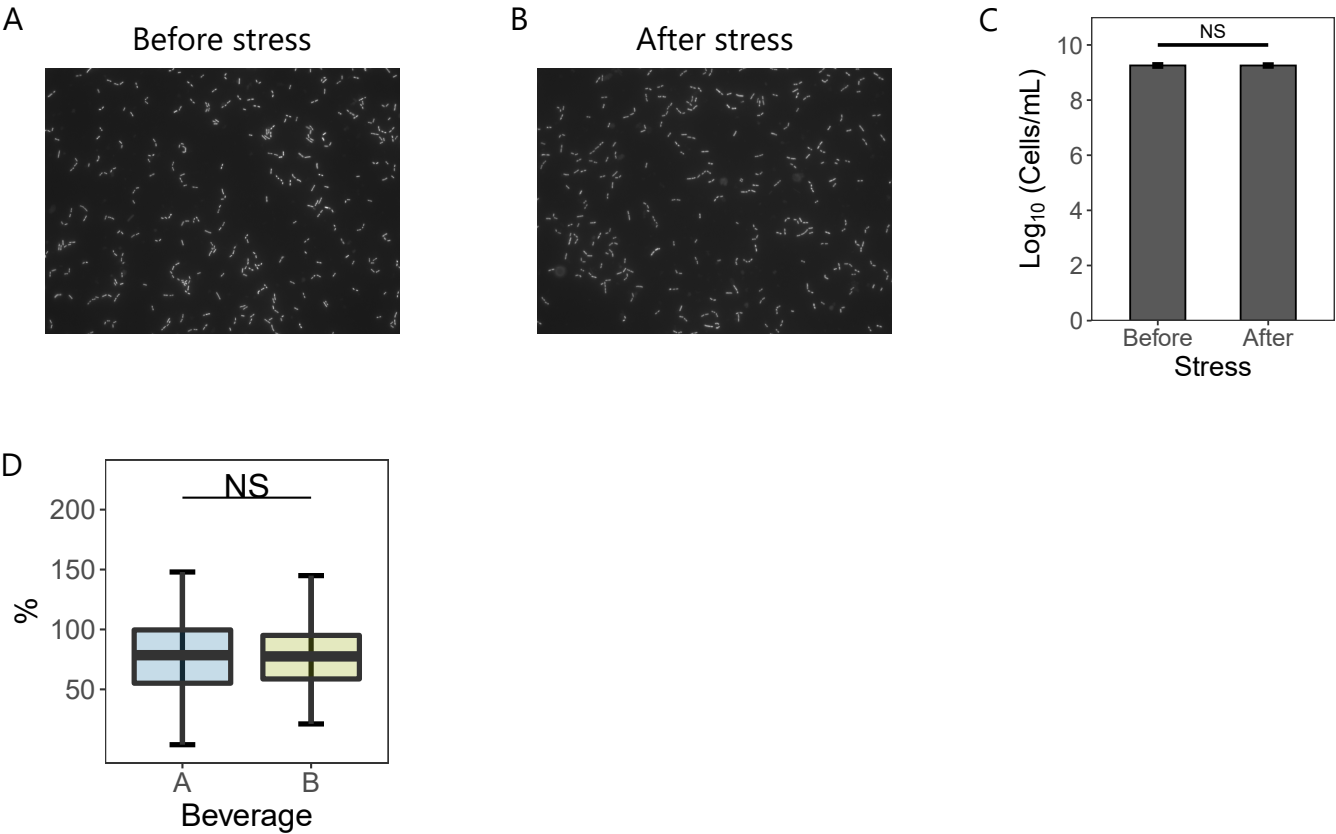

Supplement: Fig. S7 — Effect of in vitro gastrointestinal stress on the number of LcS cells and the percentage of LcS cell counts in feces after consumption of beverages. [file aem.01502-23-s0008.pdf]
